# Cationic disulfide-functionalized worm gels†
†Electronic supplementary information (ESI) available. See DOI: 10.1039/c7py01306j


**DOI:** 10.1039/c7py01306j

**Published:** 2017-09-06

**Authors:** L. P. D. Ratcliffe, K. J. Bentley, R. Wehr, N. J. Warren, B. R. Saunders, S. P. Armes

**Affiliations:** a Dainton Building , Department of Chemistry , University of Sheffield , Brook Hill , Sheffield , South Yorkshire S3 7HF , UK . Email: l.p.ratcliffe@gmail.com ; Email: s.p.armes@sheffield.ac.uk; b School of Chemical and Process Engineering , University of Leeds , Leeds , LS2 9JT , UK . Email: N.Warren@leeds.ac.uk; c School of Materials , The University of Manchester , MSS Tower , Manchester , M13 9PL , UK

## Abstract

The recent development of polymerization-induced self-assembly (PISA) has facilitated the rational synthesis of a range of diblock copolymer worms, which hitherto could only be prepared *via* traditional post-polymerization processing in dilute solution. Herein we explore a new synthetic route to aqueous dispersions of cationic disulfide-functionalized worm gels. This is achieved *via* the PISA synthesis of poly[(glycerol monomethacrylate-*stat*-glycidyl methacrylate)]-*block*-poly(2-hydroxypropyl methacrylate) (P(GMA-*stat*-GlyMA)-PHPMA) block copolymer worms *via* reversible addition–fragmentation chain transfer (RAFT) aqueous dispersion polymerization of HPMA. A water-soluble reagent, cystamine, is then reacted with the pendent epoxy groups located within the P(GMA-*stat*-GlyMA) stabilizer chains to introduce disulfide functionality, while simultaneously conferring cationic character *via* formation of secondary amine groups. Moreover, systematic variation of the cystamine/epoxy molar ratio enables either chemically cross-linked worm gels or physical (linear) primary amine-functionalized disulfide-based worm gels to be obtained. These new worm gels were characterized using gel permeation chromatography, ^1^H NMR spectroscopy, transmission electron microscopy, dynamic light scattering, aqueous electrophoresis and rheology. In principle, such hydrogels may offer enhanced mucoadhesive properties.

## Abstract 2

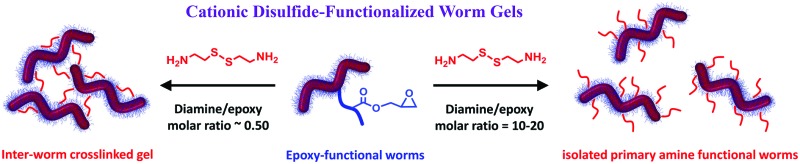
Two types of cationic disulfide diblock copolymer worm gels are prepared by reacting cystamine with epoxy groups located within the steric stabilizer chains.

## Introduction

The synthesis of well-defined functional block copolymers has been transformed over the past two decades by the development of controlled radical polymerization chemistries such as reversible addition–fragmentation chain transfer (RAFT) polymerization.^1^,^2^ In particular, the recent development of RAFT-mediated polymerization-induced self-assembly (PISA)^3^–^5^ has facilitated the rational synthesis of diblock copolymer worms,^6^–^8^ which previously had only been prepared *via* traditional post-polymerization processing in dilute solution.^9^–^12^ Recently, numerous examples of diblock copolymer worms have been reported *via* PISA syntheses conducted in water,^6^,^8^,^13^–^20^ alcohol^21^,^22^ or non-polar solvents.^23^–^27^

Relatively soft, free-standing worm gels are typically obtained, with macroscopic gelation likely to be the result of multiple physical contacts between neighbouring worms, rather than inter-worm entanglements. In particular, aqueous worm gels offer potential biomedical applications. For example, poly(glycerol monomethacrylate)-poly(2-hydroxypropyl methacrylate) (PGMA-PHPMA) diblock copolymer worm gels are thermoresponsive, undergoing degelation on cooling from 20 °C to 5 °C *via* a reversible worm-to-sphere transition. Such gels can induce stasis in human embryonic stem cells^28^ and enable solvent-free cryopreservation of red blood cells.^29^ Aqueous worm gels can also be used as 3D matrices for long-term cell culture.^30^ However, in this case it proved necessary to introduce covalent disulfide bonds between adjacent worms to preserve gel integrity over extended culture periods (*e.g.* 10–12 days). In principle, this can be achieved either by copolymerizing glycerol monomethacrylate (GMA) with a minor fraction of disulfide dimethacrylate (DSDMA) to give a disulfide-functionalized macro-CTA, or by using a disulfide-based CTA to polymerize GMA.^31^ In each case, the RAFT aqueous dispersion polymerization of HPMA resulted in block copolymer worms with disulfide functionality located within the PGMA stabilizer chains. As expected, increasing the disulfide content led to the formation of stronger gels. Such (light) crosslinking also led to lower critical gelation temperatures, because inter-worm covalent disulfide linkages hinder the worm-to-sphere transition. At a sufficiently high disulfide content, thermoresponsive behavior was no longer observed. Addition of excess tris(2-carboxyethyl)phosphine (TCEP) cleaved the disulfide bonds and so removed all inter-worm crosslinks, thus lowering the gel strength to that of a conventional non-disulfide functionalized PGMA-PHPMA worm gel.

Epoxy groups are highly reactive and strongly electrophilic, so they are readily attacked by nucleophiles such as primary amines, undergoing nucleophilic substitution to give an alkoxide anion, followed by rapid proton transfer.^32^,^33^ However, under appropriate conditions (*e.g.* neutral pH, moderate temperature) epoxides are relatively unreactive towards water,^34^ so epoxy–amine chemistry can be conducted in aqueous solution. Indeed, this approach was recently reported by Lovett and co-workers, who statistically copolymerized glycidyl methacrylate (GlyMA) with HPMA to prepare aqueous dispersions of block copolymer worms that could be subsequently core-crosslinked by reaction with 3-aminopropyltriethoxysilane (APTES) at pH 9–10.^35^ A similar strategy was also used by Chambon *et al.* to prepare the analogous covalently-stabilized vesicles using water-soluble diamines.^36^

In the present study, we explore a new and convenient synthetic route to cationic disulfide-functionalized worm gels. This is achieved *via* the PISA synthesis of poly[(glycerol monomethacrylate-*stat*-glycidyl methacrylate)]-*block*-poly(2-hydroxypropyl methacrylate) (P(GMA-*stat*-GlyMA)-PHPMA) block copolymer worms *via* RAFT aqueous dispersion polymerization. A water-soluble reagent, cystamine, is then reacted with the pendent epoxy groups located within the P(GMA-*stat*-GlyMA) stabilizer chains to introduce disulfide functionality while simultaneously conferring cationic character *via* formation of secondary amine groups. This approach has the advantage of utilizing only relatively cheap, commercially-available starting materials, rather than bespoke disulfide-based comonomers or RAFT agents. In principle, systematic variation of the cystamine/epoxy molar ratio should dictate whether either covalently cross-linked disulfide-bridged worm gels or physical primary amine-functionalized worm gels are obtained (see Fig. 4). This concept is explored herein.

## Experimental

### Materials

Glycerol monomethacrylate (GMA; 99.8%) and 2-hydroxypropyl methacrylate (HPMA, 97%) were donated by GEO Specialty Chemicals (Hythe, UK) and used without further purification. Glycidyl methacrylate (GlyMA; 97%), 4,4′-azobis(4-cyanopentanoic acid) (ACVA/V501; 99%), were purchased from Sigma-Aldrich UK and used as received. 2-Cyano-2-propyl dithiobenzoate (CPDB) was purchased from Strem Chemicals (Cambridge, UK). 2,2′-Azobis[2-(2-imidazolin-2-yl)propane]dihydrochloride (VA-044) was purchased from Wako Speciality Chemicals (Osaka, Japan) and used as received. Cystamine dihydrochloride (97%) was purchased from Acros Organics (Geel, Belgium). Deuterated methanol (CD_3_OD) was purchased from Goss Scientific (Nantwich, UK). All other solvents were HPLC-grade, purchased from Fisher Scientific (Loughborough, UK), and used as received. BioDesign Dialysis Tubing™, MWCO = 3500, was also purchased from Fisher Scientific (Loughborough, UK). Deionized water was used for all experiments.

### Synthesis of P(GMA_65_-*stat*-GlyMA_1.8_) macro-CTA *via* RAFT solution polymerization

GMA monomer (208.22 g, 1.30 mol), GlyMA monomer (5.38 g, 37.83 mmol;), CPDB RAFT agent (4.55 g, 20.58 mmol; target DP = 65), ACVA initiator (1.15 g, 4.11 mmol; CPDB/ACVA molar ratio = 5.0) and ethanol (227.4 mL) were added to a 500 mL round-bottomed flask. On stirring, this mixture formed a red 55% w/w alcoholic solution. This solution was cooled to 0 °C using an ice bath and purged with N_2_ gas for 45 min. The flask was subsequently sealed and immersed in an oil bath set at 70 °C. After 120 min, the statistical copolymerization was quenched by immersion of the flask in ice, exposing the reaction solution to air and diluting with methanol (150 mL). A final comonomer conversion of 81% was determined by ^1^H NMR analysis. The crude copolymer was purified by three consecutive precipitations into a ten-fold excess of dichloromethane. The purified copolymer was then dissolved in water and freeze-dried for 48 h to yield a pink powder. ^1^H NMR analysis of this macro-CTA indicated 65 GMA and 1.8 GlyMA units per copolymer chain, as determined by comparing aromatic proton signals arising from the dithiobenzoate end-group (7.4–8.0 ppm) to those assigned to pendent GMA units (3.4–4.2 ppm), the methacrylic copolymer backbone (1.7–2.3 ppm), and GlyMA epoxy protons (2.8–3.0 ppm). DMF GPC analysis using a refractive index detector and a series of near-monodisperse poly(methyl methacrylate) calibration standards indicated an *M*_n_ of 15 500 g mol^–1^ and an *M*_w_/*M*_n_ of 1.13.

### Synthesis of PGMA_62_ macro-CTA *via* RAFT solution polymerization

GMA monomer (208.22 g, 1.30 mol), CPDB RAFT agent (4.43 g, 20.0 mmol; target DP = 65), ACVA initiator (1.12 g, 4.00 mmol; CPDB/ACVA molar ratio = 5.0) and ethanol (221.7 mL) were added to a 500 mL round-bottomed flask. On stirring, this mixture formed a red 55% w/w alcoholic solution. This solution was cooled to 0 °C using an ice bath and purged with N_2_ gas for 45 min. The flask was subsequently sealed and immersed in an oil bath set at 70 °C. After 120 min, the polymerization was quenched by immersing the flask in ice, exposing the reaction solution to air and diluting with methanol (150 mL). A final GMA conversion of 80% was determined by ^1^H NMR analysis. The crude polymer was purified by three consecutive precipitations into a ten-fold excess of dichloromethane. The purified polymer was dissolved in water and freeze-dried for 48 h to yield a pink powder. ^1^H NMR analysis of the PGMA macro-CTA indicated a mean degree of polymerization of 62, as determined by comparing aromatic proton signals from the dithiobenzoate end-group (7.4–8.0 ppm) to proton signals on pendent GMA units (3.4–4.2 ppm) and the methacrylic backbone (1.7–2.3 ppm). DMF GPC analysis using a refractive index detector and a series of near-monodisperse poly(methyl methacrylate) calibration standards indicated an *M*_n_ of 14 600 g mol^–1^ and an *M*_w_/*M*_n_ of 1.12.

### Synthesis of P(GMA_65_-*stat*-GlyMA_1.7_)-PHPMA_*y*_ block copolymer worms by RAFT aqueous dispersion polymerization

A typical protocol for the synthesis of a P(GMA_65_-*stat*-GlyMA_1.7_)-PHPMA_140_ diblock copolymer is as follows: P(GMA_65_-*stat*-GlyMA_1.8_) macro-CTA (9.50 g, 0.87 mmol), HPMA monomer (17.63 g, 0.12 mol; target DP = 140), VA-044 initiator (70 mg, 0.22 mmol; CPDB/VA-044 molar ratio = 4.0) and water (108.72 g) were weighed into a 250 mL round-bottomed flask and purged with N_2_ for 45 min. The flask was subsequently sealed and immersed in an oil bath set at 50 °C and the reaction solution was stirred for 90 min. After quenching by immersing the flask in ice and exposing to air, ^1^H NMR analysis indicated more than 99% HPMA conversion (as judged by the complete disappearance of vinyl proton signals at 5.5 and 6.2 ppm), with no discernible loss of epoxide functionality. A series of related diblock copolymers were prepared targeting alternative PHPMA DPs (*y* = 120 or 130) using the same protocol; in all cases more than 99% conversion was achieved.

### Synthesis of PGMA_62_-PHPMA_140_ block copolymer worms by RAFT aqueous dispersion polymerization

A typical protocol for the synthesis of a PGMA_62_-PHPMA_140_ diblock copolymer is as follows: PGMA_62_ macro-CTA (0.32 g, 0.032 mmol), HPMA monomer (0.63 g, 4.41 mmol), VA-044 initiator (2.5 mg, 0.008 mmol; CPDB/VA-044 molar ratio = 4.0) and water (3.84 g) were weighed into a glass vial and purged with N_2_ for 20 min. The flask was subsequently sealed and immersed into an oil bath set at 50 °C and the reaction solution was stirred for 90 min. After quenching by immersion in an ice bath and exposure to air, ^1^H NMR analysis indicated more than 99% HPMA monomer conversion (as judged by complete disappearance of the vinyl protons at 5.5 and 6.2 ppm).

### Functionalization of P(GMA_65_-*stat*-GlyMA_1.7_)-PHPMA_*y*_ worm gels with cystamine

A typical protocol for cystamine functionalization of a P(GMA_65_-*stat*-GlyMA_1.7_)-PHPMA_140_ worm gel is as follows: a 20% w/w copolymer worm gel (1.00 g, 0.0064 mmol) was adjusted to pH 8–9 using aqueous NaOH. Cystamine (0.0492 g, 0.218 mmol; [cystamine]/[epoxide] molar ratio = 20) was then dissolved in water and adjusted to pH 8–9 using aqueous NaOH, before being added to the worm gel *via* pipette. The final copolymer concentration was then adjusted to 10% w/w using mildly alkaline water (pH 8–9) and the aqueous copolymer dispersion was stirred for 24 h at 22 °C. For ^1^H NMR studies, samples were dialyzed against deionized water for three days (changing the water twice daily), before lyophilization and dissolution in CD_3_OD.

### ^1^H NMR spectroscopy

Copolymers were dissolved in deuterated methanol (CD_3_OD) and ^1^H NMR spectra were recorded using a 400 MHz Bruker Avance spectrometer (64 scans averaged per spectrum).

### Gel permeation chromatography (GPC)

Molecular weight distributions were assessed using a DMF GPC instrument operating at 60 °C. The set-up comprised two Polymer Laboratories PL gel 5 μm Mixed-C columns and one PL polar gel 5 μm guard column connected in series to an Agilent Technologies 1260 Infinity multidetector suite and an Agilent Technologies 1260 Infinity pump injection module. The GPC eluent was HPLC-grade DMF containing 10 mM LiBr and was filtered prior to use. The flow rate was 1.0 ml min^–1^ and DMSO was used as a flow-rate marker. Calibration was conducted using a series of ten near-monodisperse poly(methyl methacrylate) standards ranging from 625 to 618 000 g mol^–1^. Chromatograms were analyzed using Varian Cirrus GPC software (version 3.3).

### Transmission electron microscopy (TEM)

0.20% w/w copolymer dispersions were prepared at 20 °C. Copper/palladium TEM grids (Agar Scientific, UK) were surface-coated in-house to produce a thin film of amorphous carbon, then plasma glow-discharged for 30 seconds to create a hydrophilic surface. Droplets of freshly-prepared aqueous copolymer dispersions (9 μL; 0.20% w/w) were placed on a hydrophilic grid for 1 min and then blotted with filter paper to remove excess solution. The deposited nanoparticles were then negatively stained with an aqueous solution of uranyl formate (9 μL; 0.75% w/w) for a further 20 seconds, then carefully blotted to remove excess stain and dried with a vacuum hose. TEM grids were imaged using a FEI Tecnai Spirit TEM instrument equipped with a Gatan 1kMS600CW CCD camera operating at 120 kV.

### Dynamic light scattering (DLS) and aqueous electrophoresis

Measurements were conducted at 25 °C using a Malvern Instruments Zetasizer Nano series instrument equipped with a 4 mW He–Ne laser (*λ* = 633 nm) and an avalanche photodiode detector. Scattered light was detected at 173°. Copolymer dispersions were diluted to 0.20% w/w. Intensity-average hydrodynamic diameters were calculated *via* the Stokes–Einstein equation. For zeta potential measurements, each aqueous worm dispersion was dialyzed against deionized water prior to analysis to remove excess cystamine, lyophilized and then redispersed at 10% w/w copolymer in mildly alkaline aqueous solution (pH 9) prior to dilution to 0.20% w/w copolymer in the presence of 1 mM KCl and the pH was adjusted using KOH as required. Zeta potentials were calculated using the Smoluchowski equation.

### Oscillatory rheology measurements

An AR-G2 stress-controlled rheometer equipped with a variable temperature Peltier plate and a 40 mL 2° aluminium cone. A solvent trap was used for all experiments, to prevent evaporation of water over the time scale of the experiment. Loss moduli (*G*′′) and storage moduli (*G*′) were measured as a function of applied strain and temperature to identify the linear viscoelastic region and determine the CGT, respectively. Temperature sweeps were conducted at a fixed angular frequency of 1.0 rad s^–1^ and a constant strain of 1.0%. In these experiments, the temperature was decreased by 1.0 °C (from 27 °C to 2 °C) between each measurement, allowing an equilibration time of 2 min in each case. Gels were prepared at 20% w/w copolymer, diluted to 10% w/w using deionized water and adjusted to pH 8–9.

## Results and discussion

**Scheme 1:**
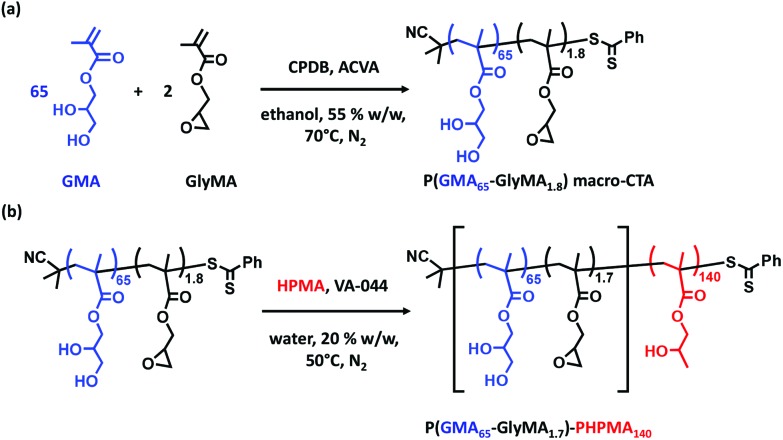
(a) Synthesis of poly(glycerol monomethacrylate-*stat*-glycidyl methacrylate) [P(GMA_65_-*stat*-GlyMA_1.8_)] macro-CTA *via* statistical copolymerization of GMA and GlyMA using 2-cyano-2-propyl benzodithioate (CPDB) RAFT agent and 4,4′-azobis(4-cyanovaleric acid) (ACVA) initiator (CTA/ACVA molar ratio = 5.0) at 55% w/w solids in ethanol at 70 °C. (b) Synthesis of poly(glycerol monomethacrylate-*stat*-glycidyl methacrylate)-poly(2-hydroxypropyl methacrylate) [P(GMA_65_-*stat*-GlyMA_1.7_)-PHPMA_140_] diblock copolymer *via* RAFT aqueous dispersion polymerization of HPMA at 50 °C and pH 6–7 using a P(GMA_65_-*stat*-GlyMA_1.8_) macro-CTA at 20% w/w solids (CTA/VA-044 = 4.0). The modest reduction in GlyMA content from 1.8 mol% to 1.7 mol% is the result of *in situ* ring-opening of the epoxide group by water, which affords GMA residues.

In initial experiments, a PGMA_62_ macro-CTA and an epoxy-functional P(GMA_65_-*stat*-GlyMA_1.8_) macro-CTA were each prepared by RAFT solution polymerization in ethanol (see Scheme 1a). Comparable DPs were targeted to assess whether the addition of GlyMA comonomer led to a reduction in RAFT control. After 2 h, ^1^H NMR studies indicated (co)monomer conversions of 80% and 81% for the PGMA_62_ and P(GMA_65_-*stat*-GlyMA_1.8_) syntheses, respectively. In the latter case, ^1^H NMR analysis suggests approximately statistical incorporation of GlyMA (data not shown). This is not unexpected given the similar chemical structures of GlyMA and GMA. After purification, DMF GPC analyses of these PGMA_62_ and P(GMA_65_-*stat*-GlyMA_1.8_) macro-CTAs indicated similar *M*_n_ values (14 600 g mol^–1^*vs.* 15 500 g mol^–1^) and comparable *M*_w_/*M*_n_ values (1.12 and 1.13 respectively), see Fig. S1a.†

**Fig. 1:**
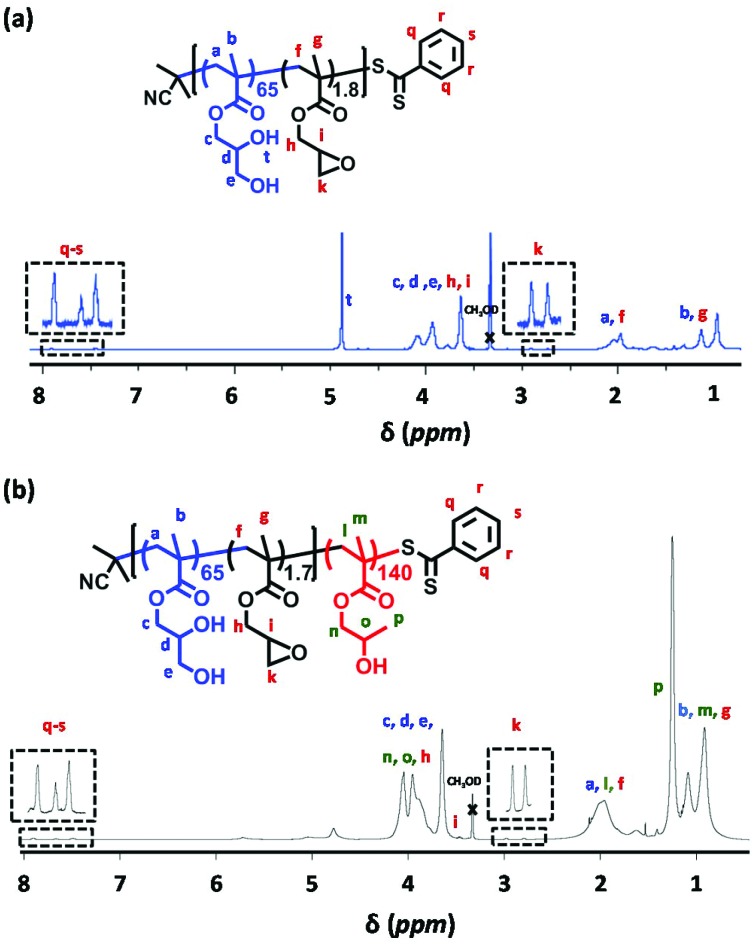
^1^H NMR spectrum in CD_3_OD recorded for (a) P(GMA_65_-*stat*-GlyMA_1.8_) and (b) P(GMA_65_-*stat*-GlyMA_1.7_)-PHPMA_140_, with expansions to show retention of the two epoxide signals at 2.8–3.0 ppm and the CTA signals at 7.4–7.9 ppm. The water signal (4.8 ppm) has been suppressed for the P(GMA_65_-*stat*-GlyMA_1.7_)-PHPMA_140_ spectrum to improve the clarity of this spectrum.

Moreover, ^1^H NMR analysis indicated that at least 92% of the epoxy groups on the GlyMA residues remained intact during RAFT solution polymerization in ethanol at 70 °C for 1.5 h (see Fig. 1). Subsequently, P(GMA_65_-*stat*-GlyMA_1.7_)-PHPMA_140_ and PGMA_62_-PHPMA_140_ worm gels were prepared *via* RAFT aqueous dispersion polymerization of HPMA in water at 20% w/w solids (see Scheme 1b). Previous work by Lovett and co-workers indicated that using an ACVA initiator at 70 °C led to significant loss of epoxy functionality during similar aqueous PISA syntheses.^35^ Thus an azo initiator with a lower 10 h half-life (VA-044) was utilized to allow the reaction temperature to be lowered to 50 °C. In addition, the reaction solution was adjusted to approximately neutral pH. These milder conditions minimized loss of epoxide functionality (∼92% GlyMA residues remained intact), while enabling very high (>99%) HPMA conversions to be achieved in both cases (see Fig. 1b).

DMF GPC analyses indicated an *M*_n_ of 37 200 g mol^–1^ (*M*_w_/*M*_n_ = 1.17) for P(GMA_65_-*stat*-GlyMA_1.7_)-PHPMA_140_ and an *M*_n_ of 36 300 g mol^–1^ (*M*_w_/*M*_n_ = 1.13) for PGMA_62_-PHPMA_140_ (see Fig. S1b†). A weak high molecular weight shoulder was observed for the former copolymer, which suggests light branching as a result of intermolecular reaction of the hydroxyl groups on the GMA residues with GlyMA residues on a second chain. There is little or no evidence for light branching for the latter copolymer, which suggests that any dimethacrylate impurities in the HPMA monomer must be negligible.^37^,^38^

Two further P(GMA_65_-*stat*-GlyMA_1.7_)-PHPMA_*y*_ diblock copolymers were also synthesized targeting *y* = 120 or 130, but free-standing gels were not obtained in these cases (see Fig. S2†). These observations illustrate that pure worms occupy relatively narrow phase space, as expected.^39^ Thus, all the following experiments in this study were conducted with the P(GMA_65_-*stat*-GlyMA_1.7_)-PHPMA_140_ copolymer.

After establishing a robust protocol for the PISA synthesis of epoxy-functional block copolymer worms, we examined their chemical derivatization using cystamine. In principle, stoichiometric quantities of this diamine with the pendent epoxy groups in the stabilizer chains should introduce inter-worm crosslinks, hence leading to stronger, less thermoresponsive gels. Alternatively, if a large excess of cystamine is employed, then only one of the two amines is likely to react with an epoxy group, leading to predominantly linear disulfide-based worms with pendent primary amine groups. Notably, cationic character is introduced in both cases, because epoxy–amine chemistry always leads to the formation of secondary amines (in addition to the pendent primary amines obtained when using excess cystamine). This clearly differentiates the present synthetic strategy from that previously reported by Warren and co-workers for the production of disulfide-functionalized worm gels.^31^ Moreover, such cationic character could be important for potential biomedical applications of these worm gels, because it is known that cationic copolymers can exhibit antimicrobial properties^40^–^45^ and stronger mucoadhesion.^46^

In view of the above considerations, three regimes were examined for cystamine derivatization: (i) sub-stoichiometric (diamine/epoxide molar ratio = 0.05–0.125), (ii) equimolar (diamine/epoxide molar ratio = 0.50) and (iii) excess (diamine/epoxide molar ratio = 1.0–20.0). One complication here is that the cystamine reagent is likely to react with the RAFT end-groups,^47^,^48^ which are present at comparable concentrations to that of the epoxy groups. In principle, access to these dithiobenzoate end-groups should be hindered because they are located within the worm cores, but in practice the PHPMA block is highly plasticized and hence rather permeable to small molecules.^6^,^49^ In principle, other possible side-reactions include (i) amidation of methacrylic ester groups and (ii) epoxide ring-opening by water (or by hydroxyl groups located on the PGMA stabilizer chains). In addition, the initially-formed secondary amine groups may react further to produce tertiary amines. Thus the equimolar conditions implied by utilizing a diamine/epoxy molar ratio of 0.50 are best considered as only approximately equimolar.

Epoxide functionality proved to be essential for functionalization with cystamine, because control experiments indicated that this reagent did not react with PGMA_62_-PHPMA_140_ (see Fig. S3†). This suggests that amidation of methacrylic ester groups is negligible under mild conditions. Cystamine derivatization studies were conducted on P(GMA_65_-*stat*-GlyMA_1.7_)-PHPMA_140_ at 22 °C to limit side reactions and pH 8–9 was chosen to ensure that a significant fraction of this reagent was present in its neutral reactive form.

**Fig. 2:**
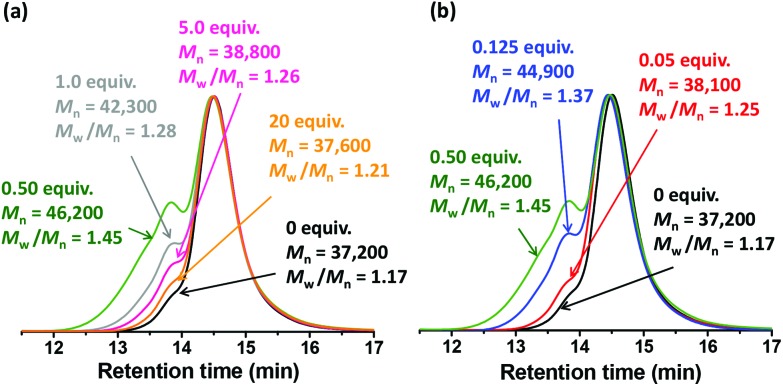
DMF GPC chromatograms obtained for the original (black traces) and cystamine-derivatized P(GMA_65_-*stat*-GlyMA_1.7_)-PHPMA_140_ worm gels. Cystamine derivation was conducted at 10% w/w copolymer for 24 h at 22 °C using (a) excess cystamine (diamine/epoxide molar ratio = 1.0, 5.0 or 20) or (b) sub-stoichiometric cystamine (diamine/epoxide molar ratio = 0.50, 0.125 or 0.05), with equimolar cystamine (diamine/epoxide molar ratio = 0.50) included for comparison in each case (see green GPC traces). [N.B. In practice, the cystamine reagent may also react with the RAFT end-groups which means that the equimolar condition is only an approximation.]

Faster reactions were observed when cystamine derivatization was conducted using more concentrated copolymer dispersions (data not shown). However, no significant change in the molecular weight distribution was observed for copolymer concentrations ranging from 5 to 20% w/w when using a diamine/epoxy molar ratio of 0.50 (see Fig. S4†). This is perhaps surprising, because higher concentrations might be expected to favour inter-worm crosslinking. In view of these preliminary observations, all further cystamine derivatizations were conducted at 10% w/w copolymer for 24 h at 22 °C, with these conditions being selected to allow efficient stirring. Fig. 2 illustrates the effect of varying the diamine/epoxy molar ratio on the final molecular weight distribution under such conditions, as judged by DMF GPC analysis. The high molecular weight shoulder observed for the P(GMA_65_-*stat*-GlyMA_1.7_)-PHPMA_140_ precursor is attributed to light branching (see above). However, the subsequent increase in *M*_n_ and *M*_w_/*M*_n_ varied dramatically depending on whether the amount of cystamine was sub-stoichiometric, approximately equimolar or in excess.

**Fig. 3:**
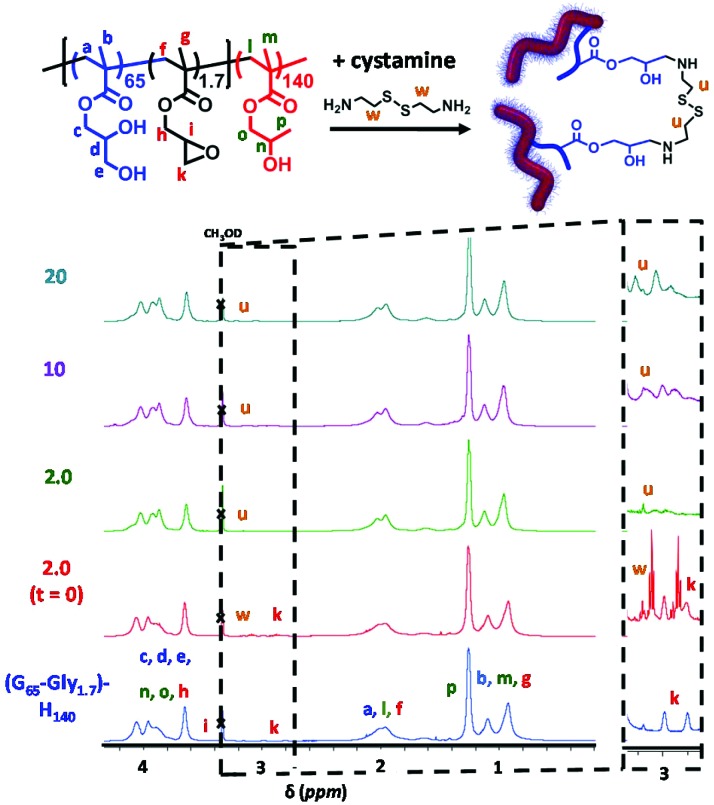
^1^H NMR spectra (CD_3_OD) obtained for cystamine-derivatized P(GMA_65_-*stat*-GlyMA_1.7_)-HPMA_140_ worm gels functionalized at 10% w/w copolymer using a diamine/epoxy molar ratio of zero, 2.0 immediately after cystamine addition (*e.g.* prior to significant epoxy ring-opening), 2.0, 10 or 20. Each worm gel was reacted at 10% w/w solids at 22 °C for 24 h. Expansion of the 2.6–3.4 ppm region is shown, indicating loss of epoxide signals and the appearance of four new broad (polymeric) cystamine signals at 3.0–3.2 ppm.

^1^H NMR studies confirmed successful derivatization of P(GMA_65_-*stat*-GlyMA_1.7_)-PHPMA_140_ using various amounts of cystamine (see Fig. 3). After allowing the epoxy–amine reaction to proceed at 22 °C for 24 h, each copolymer was dialyzed for three days against deionized water to remove any unreacted cystamine prior to lyophilization. The original epoxy proton signals at 2.8–3.0 ppm disappeared, while new broad (*i.e.* polymeric) cystamine signals appeared at 2.9–3.2 ppm; the latter signals became progressively more prominent when employing higher diamine/epoxy molar ratios.

**Fig. 4:**
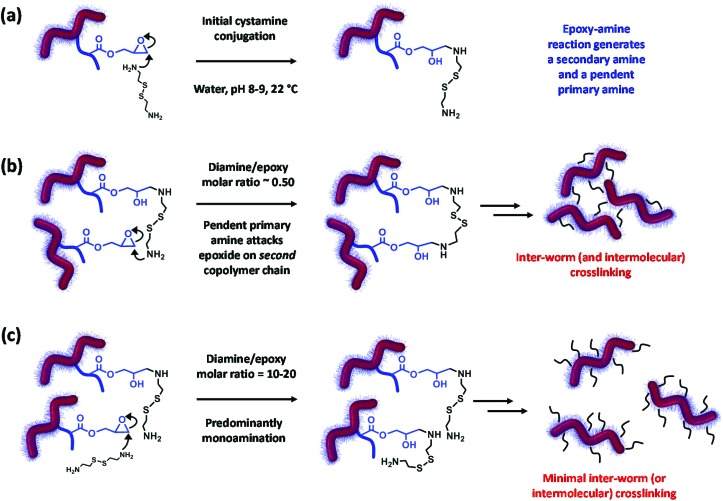
Summary of two possible scenarios for cystamine derivatization of 10% w/w P(GMA_65_-*stat*-GlyMA_1.7_)-PHPMA_140_ worm gels when systematically varying the diamine/epoxy molar ratio at 22 °C for 24 h at pH 8–9. (a) Initial reaction of cystamine with an epoxy group with concomitant proton transfer. (b) The resulting pendent primary amine can then attack an epoxide ring on another copolymer chain: if such intermolecular cross-linking occurs between adjacent worms, this leads to a covalently-crosslinked gel. (c) Using excess cystamine leads to mainly monoamination with minimal inter-worm (or inter-chain) crosslinking to produce a linear, primary amine-functionalized worm gel.

These observations can be rationalized by considering the four reaction schemes shown in Fig. 4. After the initial monoamination reaction shown in Fig. 4a, there are two likely scenarios. The largest increases in *M*_n_ and *M*_w_/*M*_n_ values are observed when using an equimolar amount of cystamine, see Fig. 4b. This was anticipated, because such conditions should lead to maximum intermolecular (and inter-worm) crosslinking. In contrast, sub-stoichiometric quantities of cystamine should only lead to light branching (not shown), while excess cystamine should result in mainly monoamination and hence minimal crosslinking with pendent primary amine groups (see Fig. 4c). In principle, after reaction of its first primary amine, the second primary amine on the cystamine could react with another epoxy group on the same copolymer chain. Again, such intra-chain reactions would not lead to any crosslinking. In practice, this latter reaction is rather unlikely in the present study because on average there are less than two epoxy groups per copolymer chain.

**Fig. 5:**
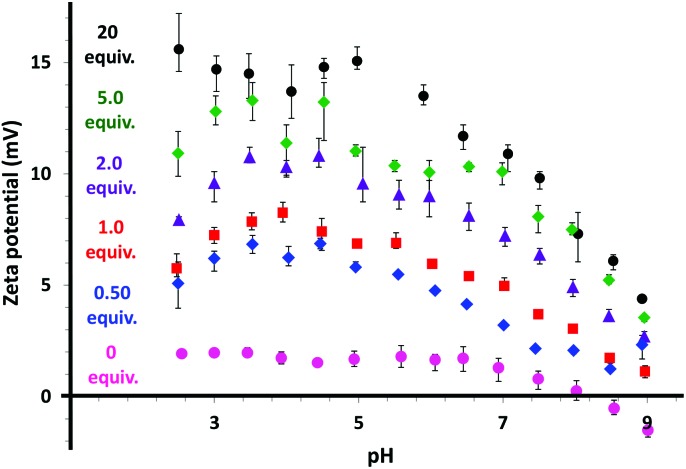
Zeta potential *vs.* pH measurements obtained for the unmodified copolymer precursor (0 equiv.) and cystamine-derivatized P(GMA_65_-*stat*-GlyMA_1.7_)-PHPMA_140_ worms prepared using diamine/epoxy molar ratios of 0.50, 1.0, 2.0, 5.0 or 20. All pH titrations were performed from high pH to low pH. Error bars indicate one standard deviation. Positive zeta potentials observed at low pH are ascribed to protonation of the secondary amine groups formed during cystamine derivatization.

Given that this epoxy–amine chemistry leads to the formation of secondary amines, aqueous electrophoresis studies were undertaken to determine whether such cystamine derivatization led to the development of cationic character for the worms. Fig. 5 shows a series of zeta potential *vs.* pH curves obtained for both the unmodified copolymer precursor (0 equiv.) and five cystamine-functionalized P(GMA_65_-*stat*-GlyMA_1.7_)-PHPMA_140_ worm gels prepared using diamine/epoxy molar ratios ranging from 0.50 to 20.

**Fig. 6:**
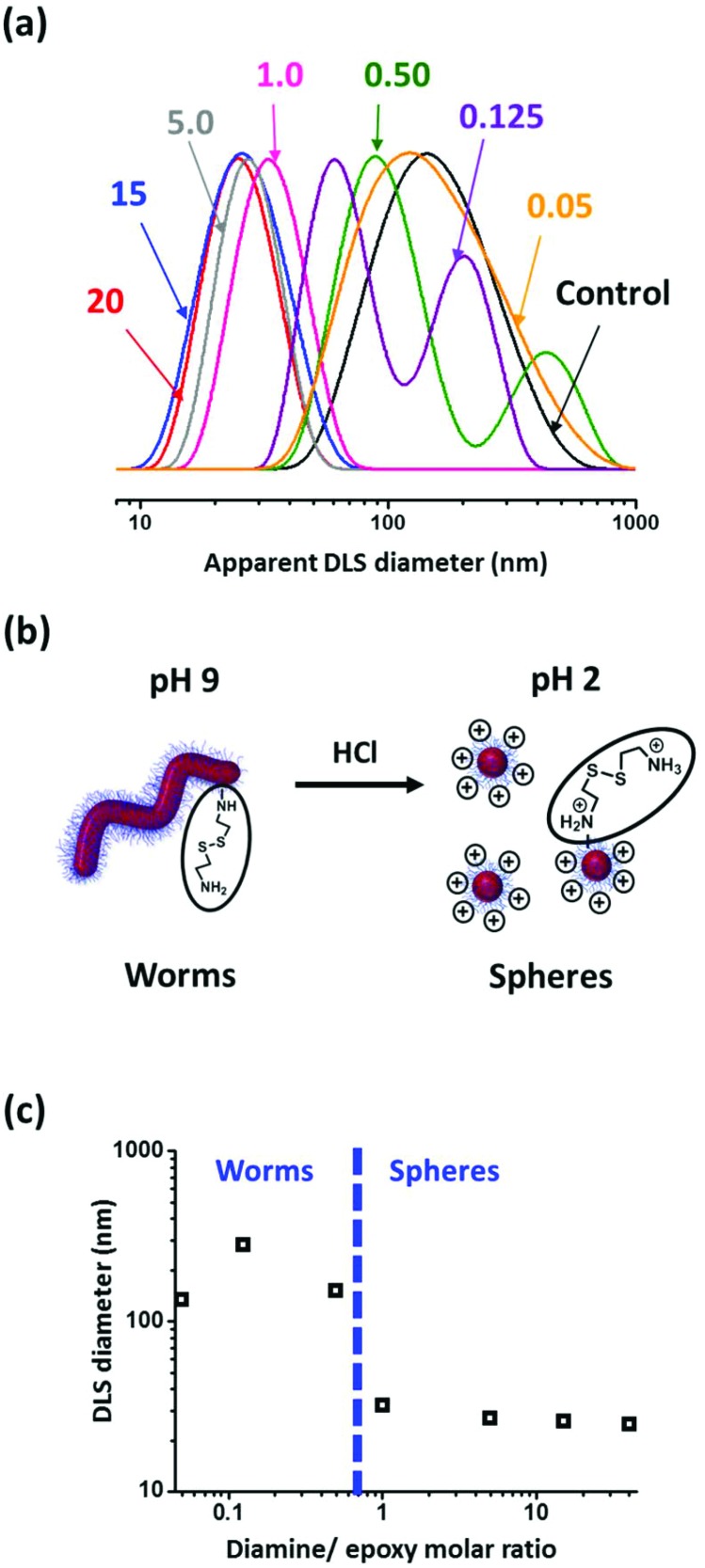
(a) DLS size distributions recorded for the precursor P(GMA_65_-*stat*-GlyMA_1.7_)-PHPMA_140_ worms (control, black curve) and cystamine-derivatized P(GMA_65_-*stat*-GlyMA_1.7_)-PHPMA_140_ worms prepared using diamine/epoxy molar ratios of 0.05, 0.125, 0.50, 1.0, 5.0, 15 or 20. DLS studies were conducted on 0.20% w/w copolymer dispersions at pH 2 immediately after dilution of a 10% w/w copolymer dispersion using dilute HCl. (b) At this low pH, worms derivatized using diamine/epoxy molar ratios ≥1.0 undergo a worm-to-sphere transition, as indicated by (c) a substantial reduction in the apparent sphere-equivalent DLS diameter. In contrast, worms prepared using diamine/epoxy molar ratios below unity remain intact.

**Fig. 7:**
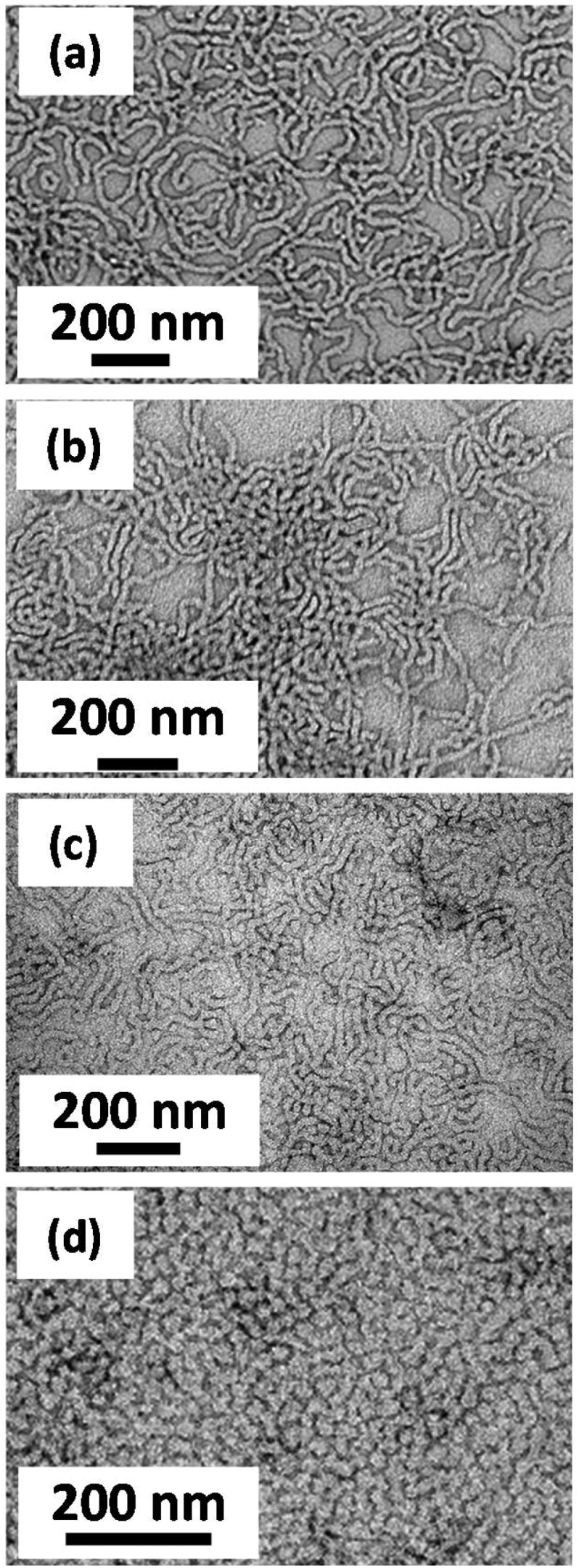
Representative TEM images recorded for: (a) PGMA_62_-PHPMA_140_ worms (control); (b) P(GMA_65_-*stat*-GlyMA_1.7_)-PHPMA_140_ worms prior to cystamine derivatization; (c) P(GMA_65_-GlyMA_1.7_)-PHPMA_140_ worms dried from acidic aqueous solution (pH 2) after derivatization using a diamine/epoxy molar ratio of 0.05; (d) mixture of P(GMA_65_-*stat*-GlyMA_1.7_)-PHPMA_140_ spheres and short worms obtained under the same conditions after derivatization using a diamine/epoxy molar ratio of 15.

Zeta potentials recorded for the P(GMA_65_-*stat*-GlyMA_1.7_)-PHPMA_140_ precursor worms range from around –1 mV at pH 9 to approximately +2 mV at pH 3. This essentially neutral character is in good agreement with previous studies of closely-related non-ionic PGMA-PHPMA diblock copolymer worms.^50^,^51^ For the cystamine-derivatized copolymers, the 1–2 secondary amine groups introduced per stabilizer chain as a result of the epoxy–amine chemistry become protonated at low pH. This confers modest cationic character, which increases with the amount of cystamine utilized for derivatization. Zeta potentials of up to +15 mV are observed at around pH 3 when employing a diamine/epoxy molar ratio of 20. Fig. 6a shows the DLS size distributions recorded at pH 2 for the precursor P(GMA_65_-*stat*-GlyMA_1.7_)-PHPMA_140_ worms (control) and a series of seven cystamine-derivatized P(GMA_65_-*stat*-GlyMA_1.7_)-PHPMA_140_ worms. The precursor worms exhibit a broad size distribution with an apparent sphere-equivalent diameter of 138 nm. This is typically characteristic^24^ of the rather polydisperse worms observed by TEM (see Fig. 7).

For cystamine derivatization performed using a diamine/epoxy molar ratio of ≤0.50, the DLS size distribution stays relatively constant at pH 2, indicating that worms remain the dominant morphology under these conditions (see Fig. 6c). However, if higher molar ratios are employed, then smaller, more uniform nanoparticles are detected and there is also a significant reduction in the scattered light intensity (data not shown): the mean sphere-equivalent diameter lies between 25 and 32 nm, suggesting a worm-to-sphere transition (or at least a substantial reduction in the mean worm contour length). Presumably, the weakly cationic character of the stabilizer block at low pH increases its relative volume fraction, leading to a reduction in the packing parameter and hence favoring spheres over worms. Similar observations were reported by Penfold and co-workers for PGMA_50_-PHPMA_140_ worms containing just a single amine group located on the stabilizer chain-ends.^51^ In the present work, this change in copolymer morphology was confirmed by TEM studies (see Fig. 7c and d).

However, worms were not reformed on returning to pH 8, as judged by DLS studies (data not shown). Such irreversibility may be related to the weakly cationic character of the spheres at around pH 7 (see Fig. 5), since this would impede the multiple sphere–sphere fusion events that are required to reform the worms. Alternatively, the relatively low copolymer concentration may be sufficient to prevent efficient sphere–sphere fusion, which becomes much less likely under such conditions. Hence further studies are warranted to establish whether the worm-to-sphere transition remains irreversible if performed at higher concentrations (*e.g.* 10% w/w copolymer).

**Fig. 8:**
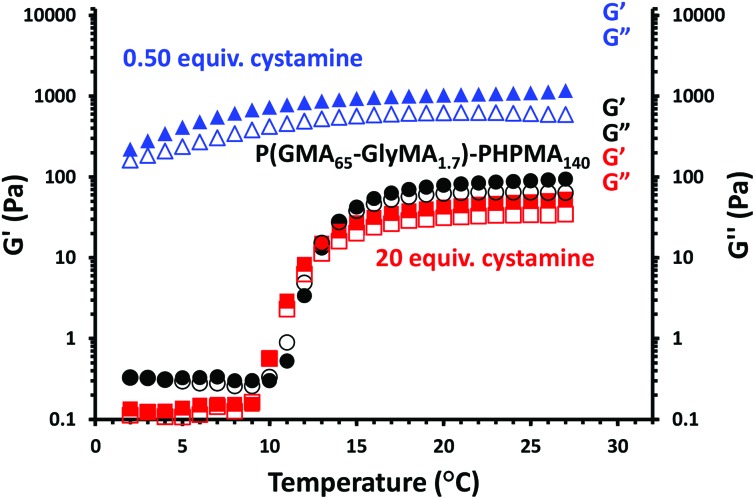
Oscillatory rheology studies showing storage moduli (*G*′, filled symbols) and loss moduli (*G*′′, open symbols) obtained on cooling 10% w/w aqueous dispersions of precursor (black circles) and cystamine-functionalized P(GMA_65_-*stat*-GlyMA_1.7_)-PHPMA_140_ worm gels, with the latter being prepared at pH 8–9 using diamine/epoxide molar ratios of either 0.50 (blue triangles) or 20 (red squares).

Preliminary rheology studies of concentrated dispersions of P(GMA_65_-*stat*-GlyMA_1.7_)-PHPMA_140_ worm gels indicate that the degree of cystamine derivatization can also significantly affect their storage moduli (*G*′) and loss moduli (*G*′′). Fig. 8 shows rheological data recorded both for the precursor P(GMA_65_-*stat*-GlyMA_1.7_)-PHPMA_140_ worm gel and after its cystamine derivatization using diamine/epoxy molar ratios of 0.50 or 20, which correspond to approximately stoichiometric conditions and a substantial excess of cystamine, respectively.

The precursor P(GMA_65_-*stat*-GlyMA_1.7_)-PHPMA_140_ worm gel undergoes a worm-to-sphere transition on cooling, which leads to *in situ* degelation. Similar observations were reported by Blanazs and co-workers for closely related PGMA_54_-PHPMA_140_ worm gels.^6^ This change in copolymer morphology is attributed to surface plasticization of the PHPMA block, which leads to a reduction in the packing parameter.^7^ Similar thermoresponsive behavior is also observed for the P(GMA_65_-*stat*-GlyMA_1.7_)-PHPMA_140_ worm gel after its derivatization using a diamine/epoxy molar ratio of 20 (see Fig. 6). The temperature at which the *G*′ and *G*′′ curves intersect corresponds the critical gelation temperature (CGT).^52^ The CGT for the cystamine-derivatized P(GMA_65_-*stat*-GlyMA_1.7_)-PHPMA_140_ worm gel is 10 °C, which is close to that observed for the copolymer precursor (CGT = 13 °C). This suggests that minimal *inter-worm* crosslinking occurs under these conditions. This is consistent with DMF GPC analysis, which indicates that minimal *intermolecular* crosslinking has occurred (see Fig. 2). In contrast, when using 0.50 equivalents of cystamine, the bulk modulus of the worm gel increases by more than an order of magnitude (*G*′ ∼10^3^ Pa) and a worm-to-sphere transition is no longer observed on cooling to 5 °C. This is in good agreement with the much higher levels of intermolecular crosslinking observed by GPC for the latter copolymer and also with observations made by Warren *et al.* regarding the physical properties of disulfide-functionalized PGMA-PHPMA worm gels.^31^

## Conclusions

Statistical copolymerization of a small amount of glycidyl methacrylate with glycerol monomethacrylate *via* RAFT solution polymerization in ethanol enables the synthesis of a near-monodisperse epoxy-functional macro-CTA. This water-soluble precursor can be chain-extended *via* RAFT aqueous dispersion polymerization of HPMA to form epoxy-functional diblock copolymer worm gels with minimal loss of epoxy groups. The epoxy groups on the steric stabilizer chains of such worms can be ring-opened with cystamine: this derivatization proceeds efficiently in aqueous solution and two types of worm gels can be obtained depending on the cystamine/epoxy molar ratio. Using a large excess of cystamine (*i.e.* a cystamine/epoxy molar ratio of 20) produces essentially linear, primary amine-functionalized worms which form a soft relatively gel that retains the thermoresponsive character of the precursor epoxy-functional worm gel. In contrast, employing a stoichiometric amount of cystamine (*i.e.* a cystamine/epoxy molar ratio of 0.50) leads to a much stronger chemically crosslinked worm gel that no longer exhibits thermoresponsive behavior. Such new hydrogels are expected to offer potential biomedical applications as next-generation mucoadhesives.

## Conflicts of interest

The authors declare no competing financial interest.

## Supplementary Material

Supplementary information
