# Supplementary material for: Cationic disulfide-functionalized worm gels
Source: Polym Chem. 2017 Sep 6;8(38):5962–71. doi: 10.1039/c7py01306j (PMC5735358; doi:10.1039/c7py01306j)
Supplement: Supplementary file 1 [file PY-008-C7PY01306J-s001.pdf]

Electronic Supplementary Material (ESI) for Polymer Chemistry.
This journal is © The Royal Society of Chemistry 2017

Supporting Information for:

Cationic Disulfide-Functionalized Worm Gels

L. P. D. Ratcliffe,[a,] K. J. Bentley,[a] R. Wehr,[a] N. J. Warren,[b,]* B. R. Saunders[c] and S. P. Armes[a,]**

[a]Dainton Building, Department of Chemistry, University of Sheffield, Brook Hill, Sheffield, South Yorkshire, S3 7HF, UK

[b]School of Chemical and Process Engineering, University of Leeds, Leeds LS2 9JT

[c]School of Materials, The University of Manchester, MSS Tower, Manchester, M13 9PL, UK

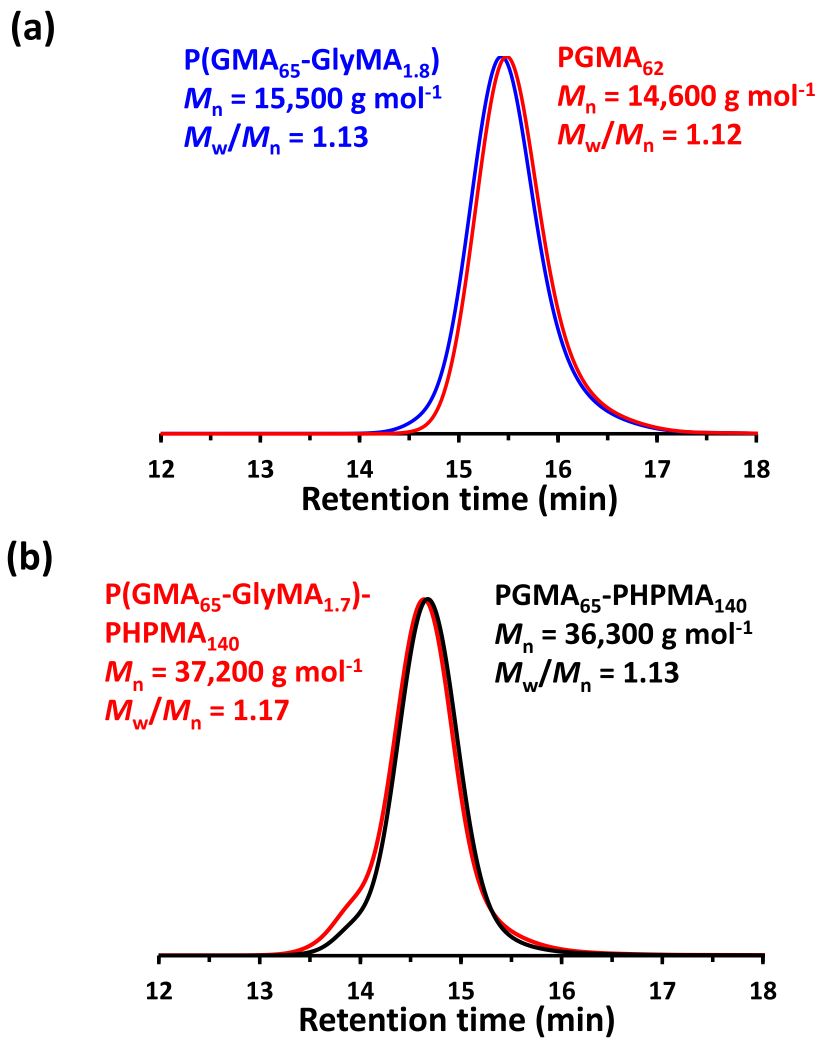

Figure S1. (a) DMF GPC chromatograms obtained for P(GMA_{65}-*stat*-$GlyMA_{1.8}$) and $PGMA_{62}$ macro-CTAs, both synthesized by RAFT solution (co)polymerization at 55 % w/w and 70 °C in ethanol, using a CTA/initiator molar ratio of 4.0. (b) DMF GPC chromatograms obtained for P(GMA_{65}-*stat*-$GlyMA_{1.7}$)-$PHPMA_{140}$ and $PGMA_{62}$-$PHPMA_{140}$ worm gels, both synthesized by RAFT aqueous dispersion polymerization of HPMA at 20 % w/w and 50 °C using a CTA/initiator molar ratio of 4.0.

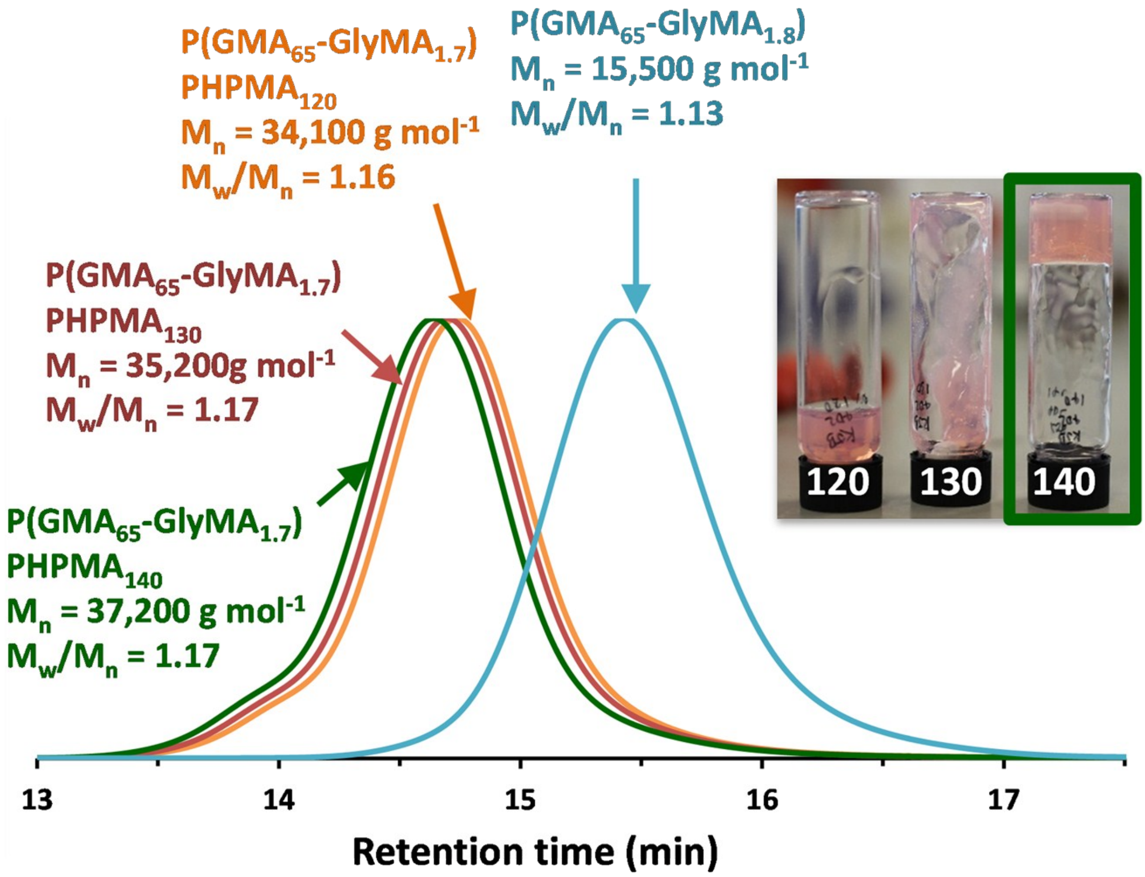

Figure S2. DMF GPC chromatograms obtained for P(GMA_{65}-*stat*-$GlyMA_{1.8}$) macro-CTA and for a series of P(GMA_{65}-*stat*-$GlyMA_{1.7}$)-$PHPMA_y$ diblock copolymers synthesized by RAFT aqueous dispersion polymerization of HPMA at 50 °C in water, at 20 % w/w solids utilizing a macro-CTA/VA-044 molar ratio of 4.0. The digital photographs of these diblock copolymers at 20 % w/w are shown, demonstrating that a free-standing gel was only formed at 22 °C for a diblock copolymer composition of P(GMA_{65}-*stat*-$GlyMA_{1.7}$)-$PHPMA_{140}$ (white text corresponds to the PHPMA DP of these copolymers). This suggests the presence of a pure worm phase, as confirmed by the representative TEM image of a 0.2 % w/w dispersion of P(GMA_{65}-*stat*-$GlyMA_{1.7}$)-$PHPMA_{140}$, diluted at 22 °C in water (see Figure 7b in the main text).

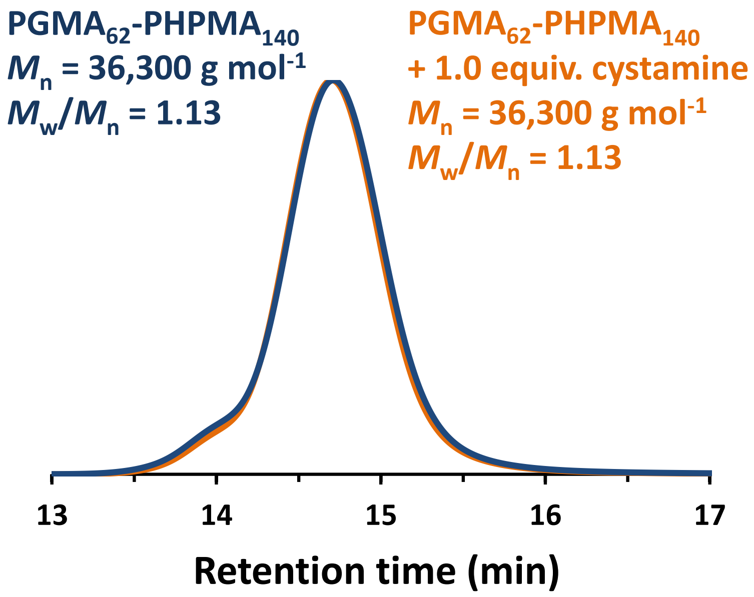

Figure S3. DMF GPC chromatograms obtained for $PGMA_{62}$-$PHPMA_{140}$, and $PGMA_{62}$-$PHPMA_{140}$ + cystamine at 10 % w/w, after stirring for 24 h at 22 °C (equivalent to cystamine/epoxide molar ratio = 1.0 for P(GMA_{65}-*stat*-$GlyMA_{1.7}$)-$HPMA_{140}$), as a control experiment.

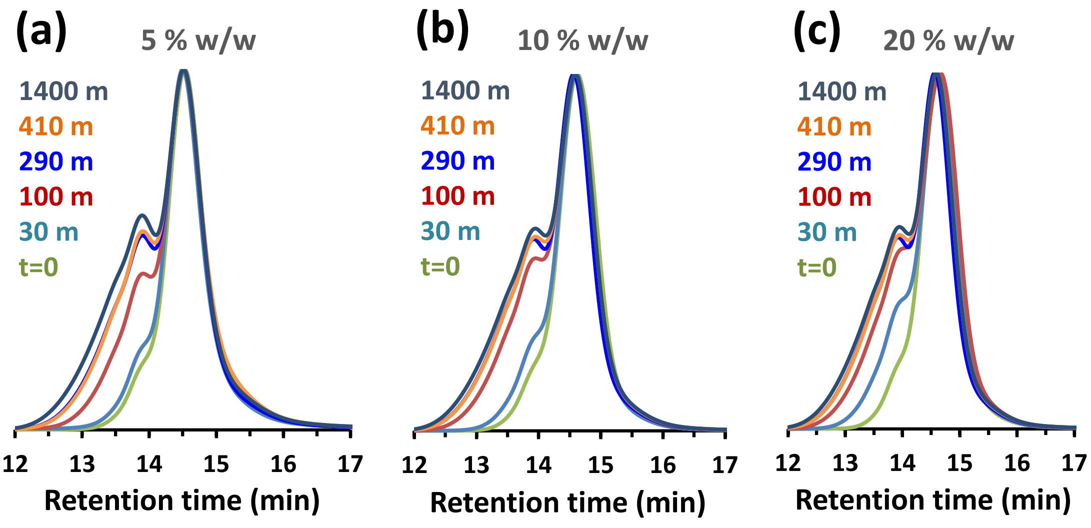

Figure S4. DMF GPC chromatograms for cystamine functionalization of P(GMA_{65}-*stat*-$GlyMA_{1.7}$)-$PHPMA_{140}$ worm gel ([cystamine]/[epoxide] = 0.50) at (a) 5 % w/w, (b) 10 % w/w, and (c) 20 % w/w solids, stirred for 24 h at 22 °C.
